# A Comparison of Cabbage Leaves *vs.* Hot and Cold Compresses in the Treatment of Breast Engorgement

**DOI:** 10.4103/0970-0218.42053

**Published:** 2008-07

**Authors:** Smriti Arora, Manju Vatsa, Vatsla Dadhwal

**Affiliations:** College of Nursing, All India Institute of Medical Sciences, Ansari Nagar, New Delhi - 110 029, India; 1 Department of Gynecology and Obstetrics, All India Institute of Medical Sciences, Ansari Nagar, New Delhi - 110 029, India

**Keywords:** Breast engorgement, hot and cold compress, treatment

## Abstract

**Objective:**

To assess and compare the efficacy of cold cabbage leaves and hot and cold compresses in the treatment of breast engorgement.

**Material and Methods:**

This was a quasi-experimental study conducted in the postnatal ward of the All India Institute of Medical Sciences (AIIMS), New Delhi. The study comprised a total of 60 mothers;30 in the experimental group and 30 in the control group. The control group received alternate hot and cold compresses and the experimental group received cold cabbage leaf treatment for relieving breast engorgement. The pre- and posttreatment scores of breast engorgement and pain were recorded. The data were analyzed using descriptive and inferential statistical methods using the statistical software STRATA.

**Results:**

Both the treatments, i.e., hot and cold compress and cabbage leaves were effective in decreasing breast engorgement and pain in postnatal mothers (*P* ≤ 0.001).Cold cabbage leaves and hot and cold compress were both equally effective in decreasing breast engorgement (*P* = 0.07), whereas hot and cold compresses were found to be more effective than cold cabbage leaves in relieving pain due to breast engorgement (*P* ≤ 0.001) in postnatal mothers.

**Conclusion:**

Cold cabbage leaves as well as alternate hot and cold compresses both can be used in the treatment of breast engorgement. Hot and cold compresses are more effective in decreasing pain than cold cabbage leaves in relieving pan due to breast engorgement.

## Introduction

Breast engorgement is a physiological condition that is characterized by painful swelling of the breasts as a result of a sudden increase in milk volume, lymphatic and vascular congestion, and interstitial edema during the first two weeks following childbirth; this condition is caused by insufficient breastfeeding and/or obstruction in milk ducts. Breast pain during breastfeeding is a common problem that interferes with successful breastfeeding leading to exclusive abandonment of breastfeeding.(1) Over the years, numerous strategies for the treatment of this problem have been employed such as kangaroo care, fluid limitation, binding the breasts or wearing a tight brassiere, hot and cold compresses, and application of cabbage leaves. Very few researches have been conducted tomonitor the effect of cabbage leaves on breast engorgement with inconclusive and conflicting results. A study on the efficacy of cabbage leaves can contribute to provide evidence for introducing this intervention in clinical practice; thus, we conducted the present study.

## Materials and Methods

We carried out a quasi-experimental study, using a time series, nonequivalent control group design, with multiple institutions of treatment in the postnatal ward at All India Institute of Medical Sciences (AIIMS), New Delhi; we conducted the study in 60 subjects between May 2006 and December 2006.

## Inclusion criteria

- Postnatal mothers with breast engorgement
- Willingness to participate in the study

## Exclusion criteria

- Mothers with allergy to sulfa drugs and cabbage
- Mothers with soft breasts; mothers receiving lactation suppressants
- Mothers with infection in the breasts, breast abscess, mastitis, broken skin of breasts, bleeding or cracked nipples

The subjects were enrolled based on the inclusion and exclusion criteria, and informed consent was then obtained from them. The identification data and obstetric characteristics of each subject were recorded in a validated subject data sheet. The study was conducted in two phases. In the first phase, first 30 mothers in the control group were administered alternate hot and cold compresses. Alternate warm moist sponge cloths and cold compresses were applied to the engorged breasts; the cloths were replaced frequently after 1–2 min. This process was continued for 20 min. The temperature of water for hot compress ranged between 43°C and 46°C, and that for cold compress ranged between 10°C and 18°C as assessed by alotion thermometer. After the completion of the first phase, the next 30 mothers in the experimental group were administered cold cabbage leaf treatment for relieving breast engorgement. Cabbage leaves were refrigerated in the freezer for approximately 20–30 min prior to the procedure. Cold cabbage leaves were placed inside the women's brassiere for 30 min. Both the treatments were performed three times a day for two continuous days. This method was applied six times on each subject. The duration of each intervention was 30 min. The pre- and posttreatment scores of breast engorgement and pain were recorded after each treatment session. Breast engorgement was measured using a six-point breast engorgement scale,(2) and the pain score was assessed using a numerical rating pain scale. The data obtained was processed in MS Excel sheet. The statistical analysis was performed using the software STRATA.

## Results

The two groups were homogeneous with regard to alldemographic and obstetric variables as analyzed by chi-square and Fisher's exact test except for breastfeeding for which adjusted analysis using generalized estimating equations (GEE) was performed. By using the student,s *t* test, no difference was noted between the groups with regard to the pretreatment scores of breast engorgement and pain. GEE was used to compare the correlated responses for the posttreatment scores for both the outcome variables between the two groups and to compare the pre- and posttreatment scores within the groups. Both the treatments, i.e., hot and cold compress and cabbage leaves, were effective in decreasing breast engorgement and pain in postnatal mothers (*P* ≤ 0.001) as shown in Table 1. Both the cold cabbage leaves and hot and cold compress procedures were equally effective in decreasing breast engorgement (*P* = 0.07) as shown in Table 2. Hot and cold compresses were found to be more effective than cold cabbage leaves in relieving pain due to breast engorgement (*P* ≤ 0.001) in postnatal mothers.

**Table 1:** Comparison of the pre- and posttreatment scores for breast engorgement and pain in both groups

	Pretreatment mean (SE) N = 30	Posttreatment mean (SE) N = 30	*P*
Control group			
Breast engorgement score	5.03 (.72)	2.97 (0.2)	<0.001***
Pain score	6.1 (1.5)	0.51 (0.4)	<0.001***
Experimental group			
Breast engorgement score	5.17 (0.7)	3.02 (0.2)	<0.001***
Pain score	6.4 (1.2)	3.45 (0.4)	<0.001***

*** P values are significant at 0.001 level

**Table 2:** Comparison of the posttreatment breast engorgement scores in postnatal mothers in both groups

No. of application	Control group mean (SE) (N = 30)	Experimental group mean (SE) (N = 30)	Mean difference	95% C.I.	*P*
1	5.14 (0.21)	4.22 (0.16)	0.18	30–0.48	0.29
2	3.84 (0.19)	4.06 (0.16)	0.22	0.13–0.56	0.23
3	3.51 (0.19)	3.92 (0.16)	0.42	0.05–0.2	0.03*
4	3.743 (0.21)	4.26 (0.17)	0.52	11–0.91	0.01†
5	3.18 (0.18)	3.23 (0.18)	0.1	0.22–0.33	0.73
6	2.97 (0.17)	3.03 (0.13)	0.1	0.17–0.27	0.6

* *P* values are significant at 0.05 level

† *P* values are significant at 0.01 level

## Discussion

This study was supported by the findings of Snowden HM *et al.*(3) who reviewed research studies to determine the effects of several interventions to relieve symptoms of breast engorgement among breastfeeding women and found that cabbage leaves were effective in the treatment of this painful condition. Cabbage leaves were preferred by the mothers. The advantage of using cabbage leaves is its low cost and convenience as compared to other medical regimens.

Roberts KL *et al.*(4) also compared the efficacy of cabbage leaf extract with that of a placebo in the treatment of breast engorgement in lactating women; they concluded that both the groups received equal relief from the discomfort and the hardness in breast tissue decreased substantially. The present study also supports the findings of Hill PD and Humenick SS(2) who reported that the type of delivery and parity are not a critical variable in predicting breast engorgement.

## Conclusion

Cold cabbage leaves as well as alternate hot and cold compresses both can be used in the treatment of breast engorgement. Hot and cold compresses are more effective than cold cabbage leaves in relieving pain due to breast engorgement.
